# Low bacterial community diversity in two introduced aphid pests revealed with 16S rRNA amplicon sequencing

**DOI:** 10.7717/peerj.4725

**Published:** 2018-05-07

**Authors:** Francisca Zepeda-Paulo, Sebastían Ortiz-Martínez, Andrea X. Silva, Blas Lavandero

**Affiliations:** 1 Laboratorio de Control Biológico/Instituto de Ciencias Biológicas, Universidad de Talca, Talca, Chile; 2 AUSTRAL-omics Facultad de Ciencias, Universidad Austral de Chile, Valdivia, Chile

**Keywords:** *Sitobion avenae*, *Hamiltonella defensa*, *Pseudomonas*, *Regiella insecticola*, Aphids, Bacterial community, Endosymbionts, Phytopathogens, *Rhopalosiphum padi*, 16S rRNA amplicon sequencing

## Abstract

Bacterial endosymbionts that produce important phenotypic effects on their hosts are common among plant sap-sucking insects. Aphids have become a model system of insect-symbiont interactions. However, endosymbiont research has focused on a few aphid species, making it necessary to make greater efforts to other aphid species through different regions, in order to have a better understanding of the role of endosymbionts in aphids as a group. Aphid endosymbionts have frequently been studied by PCR-based techniques, using species-specific primers, nevertheless this approach may omit other non-target bacteria cohabiting a particular host species. Advances in high-throughput sequencing technologies are complementing our knowledge of microbial communities by allowing us the study of whole microbiome of different organisms. We used a 16S rRNA amplicon sequencing approach to study the microbiome of aphids in order to describe the bacterial community diversity in introduced populations of the cereal aphids, *Sitobion avenae* and *Rhopalosiphum padi* in Chile (South America). An absence of secondary endosymbionts and two common secondary endosymbionts of aphids were found in the aphids *R. padi* and *S. avenae,* respectively. Of those endosymbionts, *Regiella insecticola* was the dominant secondary endosymbiont among the aphid samples. In addition, the presence of a previously unidentified bacterial species closely related to a phytopathogenic Pseudomonad species was detected. We discuss these results in relation to the bacterial endosymbiont diversity found in other regions of the native and introduced range of *S. avenae* and *R. padi*. A similar endosymbiont diversity has been reported for both aphid species in their native range. However, variation in the secondary endosymbiont infection could be observed among the introduced and native populations of the aphid *S. avenae,* indicating that aphid-endosymbiont associations can vary across the geographic range of an aphid species. In addition, we discuss the potential role of aphids as vectors and/or alternative hosts of phytopathogenic bacteria.

## Introduction

Associations between bacterial endosymbionts and insects are widespread in nature (Gibson & Hunter, 2010). The microbial community inhabiting insects can be as diverse as the symbiotic associations that they maintain with their host insects. Mutualistic, pathogenic, and commensal relationships can take place concurrently and can significantly influence the insect host ecology (Toft & Andersson, 2010). For instance, ancient mutualistic relationships with primary or obligate bacterial endosymbionts that provide missing essential amino acids to phloem-based diets are common among plant sap-sucking insects (e.g., psyllids, whiteflies, mealybugs and aphids) (Baumann, 2005). Primary endosymbionts are usually found among the Betaproteobacteria and Gammaproteobacteria subgroups (Toft & Andersson, 2010). Contrary to primary endosymbionts, secondary or facultative endosymbiotic bacteria are not essential for host survival and reproduction and they are mainly found among the Alphaproteobacteria, Gammaproteobacteria (especially *Enterobacteriaceae*) and Bacteroidetes (Baumann, 2005; Moran, McCutcheon & Nakabachi, 2008). However, secondary endosymbionts may produce ecologically important phenotypic effects on their insect hosts. Specifically, they can establish facultative mutualistic associations with insects thus conferring beneficial traits such as protection against natural enemies (review by Oliver et al., 2010; Jaenike et al., 2010; Jiggins & Hurst, 2011), or they can establish parasitic associations that have deleterious effects on host fitness (Werren, Baldo & Clark, 2008).

Aphids (Hemiptera: Aphididae) are phloem-feeding insects that reproduce by cyclical parthenogenesis (clonal) (Simon, Rispe & Sunnucks, 2002). They represent serious pests by reducing crop yields and quality, and can act as vectors of phytopathogenic viruses and bacteria (Dedryver, Le Ralec & Fabre, 2010; Ng & Perry, 2004; Nadarasah & Stavrinides, 2011). At least 15 aphid species are considered global crop pests of major agricultural importance (including the grain aphid *Sitobion avenae*, bird cherry-oat aphid *Rhopalosiphum padi* and pea aphid *Acyrthosiphon pisum*), of which the majority are of Palaearctic origin (Eurasia) (Van Emden & Harrington, 2017). Symbiotic bacteria have been well studied in this insect group, becoming a model system of the insect-symbiont interactions (Oliver, Smith & Russell, 2014). Aphids have a well-known obligate nutritional relationship with the primary endosymbiont *Buchnera aphidicola,* which confers essential nutrients to the aphid host (Douglas, 1998). At least nine common secondary endosymbionts have been reported among aphid species, including six Gammaproteobacteria; *Hamiltonella defensa, Serratia symbiotica*, *Regiella insecticola,* PAXS (Pea aphid X-type symbiont), *Rickettsiella viridis* and *Arsenophonus* sp., and two Alphaproteobacteria of the genera *Wolbachia* and *Rickettsia*, as well *Spiroplasma* from Mollicutes (reviewed in Zytynska & Weisser, 2016). These secondary endosymbionts have diverse effects on the aphid phenotype, such as conferring protection against natural enemies (parasitoids and fungal pathogens) (Oliver et al., 2003; Oliver, Moran & Hunter, 2005; Vorburger, Gehrer & Rodriguez, 2009; Scarborough, Ferrari & Godfray, 2005; Parker et al., 2013), providing resistance to heat stress (Montllor, Maxmen & Purcell, 2002), influencing insect-plant interactions (Tsuchida, Koga & Fukatsu, 2004; Tsuchida et al., 2011; Ferrari, Scarborough & Godfray, 2007), as well as manipulating aphid reproduction (Simon et al., 2011). These heritable bacterial endosymbionts are mainly maintained in aphid populations through vertical transmission (i.e., maternal) and to a lesser extent by horizontal transmission (e.g., sexual) (Vorburger, 2014; Peccoud et al., 2014). Although, the aphid–endosymbiont interactions have received considerable attention, much of this research has been focused in the model pea aphid, *A. pisum*. Accordingly, there is a lack of data for some aphid species across different regions particularly at the continental scale (e.g., South America) (Zytynska & Weisser, 2016). Therefore, it is necessary to make greater efforts to other aphid species in order to have a better understanding of the role of endosymbionts in aphids as a group. In addition, aphid endosymbionts have frequently been studied by PCR-based approaches, using species-specific primers. In spite of increasing the ease of testing for specific symbionts, and being useful for detecting target endosymbiont groups, this approach may omit other non-target bacteria cohabiting a particular host species. Regarding this, advances in high-throughput sequencing technologies are now complementing our previous knowledge of microbial endosymbiont communities (Riesenfeld, Schloss & Handelsman, 2004). A greater understanding of the microbiome of aphid species through next-generation sequencing could allow the identification of novel bacterial associations and their potential effects on the ecology and phenotype of aphid species. Such knowledge could be instrumental for understanding the role of the bacterial interactions on the invasive potential of economically important aphid species.

We used a 16S rRNA amplicon sequencing approach to study the microbiome of aphids, in order to describe the bacterial community diversity in introduced populations of the cereal aphids, *Sitobion avenae* and *Rhopalosiphum padi* in Chile (South America). Then we discuss whether the bacterial community diversity found in these introduced populations of cereal aphids is similar to the previously estimated in native populations of these aphid species (Europe).

## Materials and Methods

### Sample collection and DNA extraction

A total of 80 individuals of the aphid *S. avenae* and 52 individuals of the aphid *R. padi* were collected from oat (*Avena sativa*) and wheat (*Triticum aestivum*) crops in two different agroclimatic regions (Maule and Los Ríos regions) in Chile (Table 1). In addition, the field experiments performed in this study were approved for Ethical scientific committee of the Universidad de Talca in Chile (FONDECYT project 3140299). DNA extraction was individually performed for each aphid specimen using the “Salting out” method described by Sunnucks & Hales (1996). The quantification and quality of the extracted DNA was examined by absorbance using Infinite 200 PRO NanoQuant (TECAN) and by electrophoresis in 0.8% agarose gels. Each individual DNA extraction was normalized to a concentration of 5 ng/ul and kept at −20 °C until later 16 S library preparation.

**Table 1:** Summary of collection details and 16S rRNA gene sequencing results for aphid samples. Host plant, locality, date, total number of reads, and Shannon diversity index for each sample of *S. avenae* (SA-1, SA-2, SA-3 and SA-4) and *R. padi* (RP-1, RP-2, RP-3, RP-4, RP-5 and RP-6).

Sample ID	Host plant	Locality	Date	Numbers of reads after filtering	Shannon index
SA-1	Oat	Los Ríos	10/2014	165,703	2.39
SA-2	Wheat	Los Ríos	11/2014	317,806	1.13
SA-3	Wheat	Maule	10–12/2013	500,906	1.2
SA-4	Wheat	Maule	10–12/2013	343,371	1.96
Total				1,327,786	1.51 (0.61)
RP-1	Wheat	Maule	10/2013	210,118	0.16
RP-2	Wheat	Maule	10/2013	372,013	0.06
RP-3	Wheat	Maule	10/2013	402,514	0.06
RP-4	Wheat	Maule	10/2013	351,336	0.06
RP-5	Wheat	Maule	10/2013	556,558	0.07
RP-6	Wheat	Maule	10/2013	203,033	0.06
Total				2,095,602	0.07 (0.04)

### 16S rRNA amplicon sequencing library preparation

In order to produce DNA pools that represent the genetic diversity of aphids from different species, locations and host-plants; four DNA pools of 20 *S. avenae* aphids and six DNA pools of 9 *R. padi* aphids were used (Table 1). Pools of the genomic DNA were generated in two steps using the Illumina MiSeq protocol for 16S amplicon sequencing (https://support.illumina.com/downloads/). The V3 and V4 variable regions of the 16S ribosomal RNA gene were amplified using the primer pair S-D-Bact-0341-b-S-17 and S-D-Bact-0785-a-A-21 (Klindworth et al., 2012); these regions have a total length of approximately 460 bp. Specific Illumina Adapter sequences were added to the 5′ region of the forward (5′-TCGTCGGCAGCGTCAGATGTGTATAAGAGACAG) and reverse primers (5′-GTCTCGTGGGCTCGGAGATGTGTATAAGAGACAG). PCR reactions were performed in a volume of 15 µl; 1.5 µl of normalized DNA (5 ng/µl), each primer at 0.2 µM plus 7.5 µl of 2x KAPA Hifi HotStart Ready Mix (KAPA Biosystems, Wilmington, MA, USA). The PCR program consisted of initial denaturation at 95 °C for 3 min, followed by 25 cycles of:  95 °C for 30 s, 55 °C for 30 s and 72 °C for 30 s, and then a final extension of 72 °C for 5 min. The amplicons were purified using a PCR clean-up protocol including 10 µl of PCR product, 8 µl of AMpureXP (Beckman Coulter, Brea, CA, USA), and 200 µl of 80% ethanol. The mixture was incubated on a magnetic stand and diluted in 22.5 µl of Tris (10 mM), pH 8.5. The expected size of PCR amplicons was verified using the DNF-900 Kit for Fragment Bioanalyzer (Advanced Analytical Technologies, Inc., Ankeny, IA, USA); the quantity of amplicons was estimated by fluorescence using the Quant-iT PicoGreen dsDNA Assay kit (Invitrogen, Carlsbad, CA, USA) and a HOEFER DQ300 fluorometer (Hoefer Inc., Holliston, MA, USA). Each DNA pool was constructed by mixing 5 µl of each of the amplicons (Table 1). Then DNA pools were subjected to a second PCR where dual indices and Illumina sequencing adapters were attached using a NEXTERA XT index Kit (Illumina, San Diego, CA, USA). This second PCR was conducted in a total volume of 50 µl which contained 5 µl of each pooled DNA, 5 µl of each Nextera XT index primer, 25 µl of 2x KAPA Hifi HotStart Ready Mix, and 10 µl of PCR grade water. PCR program consisted of initial denaturation at 95 °C for 3 min, followed by eight cycles of: 95 °C for 30 s, 55 °C for 30 s and 72 °C for 30 s, and 72 °C for 5 min. The PCR product was corroborated using a Fragment Analyzer and the DNF 479 kit. Finally, each DNA pool was normalized to a concentration of 4 nM and then pooled. The mix DNA pool was prepared for sequencing using the Denature and Dilute Libraries Guide. Paired-end sequencing was performed using the Miseq Reagent Kit v3 (2 × 300 cycles) on the MiSeq Illumina sequencing platform in the AUSTRAL-*omics* Core-Facility (Facultad de Ciencias, Universidad Austral de Chile).

### Data analysis

Removal of adapters and quality filtering of the data were conducted using the Trimmomatic and PRINSEQ software (Bolger, Lohse & Usadel, 2014; Schmieder & Edwards, 2011). To assemble the overlapping Illumina Paired-end reads PANDAseq was used (Masella et al., 2012). In order to determine operational taxonomic units (OTUs), sequences sharing 97% identity were assembled as suggested by Kunin et al. (2010); this was done using the software QIIME (Caporaso et al., 2010). The OTUs were aligned using the GreenGenes database (http://greengenes.lbl.gov). Bacterial diversity was studied using the Shannon diversity index calculated for each DNA pool. The relative abundance of each OTU was estimated by examining the number of reads for each sequence and each sample as recommended by Jousselin et al. (2016). Taking into account that bacterial DNA contaminants can be commonly found in DNA extraction kits and other laboratory reagents or could enter samples during analysis (Salter et al., 2014), reads from taxa accounting for <1% of all the reads of a given sample were excluded from the data analysis (“unrepresented reads”). Regarding this, Jousselin et al. (2016) found that the removal of low frequency sequences (<1%) excluded the most DNA contaminants allowing for increased repeatability and reliability of results. They showed that by using this method, DNA contaminants have little impact on the analysis of aphid endosymbionts when using 16S rRNA Illumina sequencing. While, reads for which significant BLAST hits with known taxon could not be found are detailed as “unassigned reads”.

### Identifying *Pseudomonas* species by 16S Sanger sequencing

From the 16S rRNA amplicon sequencing, a species of *Pseudomonas* was encountered (see ‘Results’). In order to characterize the *Pseudomonas* species from the 16S rRNA sequences identified, a portion of the 16S and 23S ribosomal genes (∼1,500 bp) was amplified and sequenced in 20 aphids collect from field and used to prepare sample SA-1 (Table 1); this was done using the universal bacterial primers 10F and 35R (Sandström et al., 2001; Russell & Moran, 2005). These primers were selected because they target the intergenic spacer between the 16S and 23S genes, which can be used to avoid amplifying the aphid primary endosymbiont, *B. aphidicola* as both genes are not contiguous in this endosymbiont (Russell & Moran, 2005).

The PCR reactions were performed in a total volume of 25 µl including; 2.5 µl of 10 × buffer, 0.2 mM dNTP’s, 2 mM MgCl_2_, 0.3 µl of Taq (5 U/µl), each primer at 0.5 uM, and 3 µl of DNA (10 ng/µl). The PCR conditions consisted of initial denaturation at 94 °C for 5 min, followed by 35 cycles of 94 °C for 40 s, 57 °C for 40 s and 72 °C for 45 min and a final extension at 72 °C for 7 min. The resulting amplicons were sequenced in an ABI PRISM® 310 Genetic Analyzer (Applied Biosystems, Foster City, CA, USA). The alignment of the sequences with known 16S rRNA sequences from species of the genus *Pseudomonas* was conducted in Geneious v.8.1 (Drummond et al., 2011). All sequences of the genus *Pseudomonas* were obtained from GenBank, including sequences from the seven *Pseudomonas* clusters reported by Anzai et al. (2000): “*Pseudomonas syringae* group”, “*Pseudomonas chlororaphis* group”, “*Pseudomonas fluorescens* group”, “*Pseudomonas putida* group”, “*Pseudomonas stutzeri* group”, “*Pseudomonas aeruginosa* group”, and “*Pseudomonas pertucinogena* group” (Data S1). A phylogenetic tree of the *Pseudomonas* sequences was constructed using the HKY genetic distance model and the neighbor-joining method implemented in Geneious v.8.11 (Drummond et al., 2011). Branch significance was calculated using bootstrap values of 1,000 replications.

## Results

### 16S rRNA amplicon sequencing

**Figure 1:**
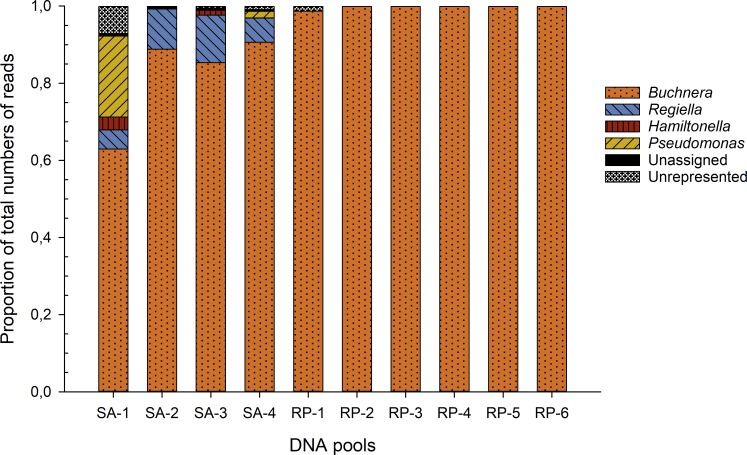
Summary of 16S rRNA gene sequencing-based taxonomic assignments. Proportion of taxa assignments for each DNA pool of *S. avenae* (SA-1, SA-2, SA-3 and SA-4) and *R. padi* (RP-1, RP-2, RP-3, RP-4, RP-5 and RP-6). The proportion of sequences assigned to <1% of the total reads per sample are identified as ‘unrepresented’, and sequences that did not cluster with any known sequences are identified as ‘unassigned’.

A total of 1,327,786 reads were obtained after filtering the four DNA pools of the aphid *S. avenae* (SA-1, SA-2, SA-3 and SA-4) (Table 1). The mean Shannon diversity index was 1.51 (SD = 0.61) and ranged from 1.13 to 2.39 for the aphid *S. avenae* (Table 1). Of the total reads for *S. avenae*, 98% were classified as Gammaproteobacteria and included mostly bacteria from the Enterobacteriaceae (94.7% of the total reads) (*Buchnera aphidicola*, *Regiella insecticola* and *Hamiltonella defensa*) and to a lesser extent from the Pseudomonadaceae (*Pseudomonas*) families (3.3% of the total reads). The Gammaproteobacteria, the aphid primary endosymbiont, *Buchnera aphidicola*, was the most common endosymbiont in the four samples (84.4% of the total reads). The second most common taxon was the aphid secondary endosymbiont *R. insecticola* (9.3% of the total reads), which was also found in all studied samples (Fig. 1). Whilst another well-known aphid endosymbiont, *H. defensa,* represented an average of 0.9% of the total reads in two of the four samples studied (SA-1 and SA-3) (Fig. 1 and Data S2). *Pseudomonas* sp. sequences were well represented in two aphid samples (SA-1 and SA-4), making up an average of 3.3% of the total reads (Fig. 1 and Data S2). Unrepresented reads (i.e., reads of taxa accounting for <1% of all the reads; see methods) were found in an average of 1.5% of the total reads. Also, the four DNA pools of *S. avenae* had a low proportion of unassigned reads (i.e., reads for which no significant BLAST hits with known taxon were found); unassigned reads ranged from 0.5% to 0.6% (average of 0.5% of the total of reads).

For the aphid *R. padi,* a total of 2,095,602 reads were obtained from the six DNA pools analyzed (RP-1, RP-2, RP-3, RP-4, RP-5 and RP-6) (Table 1). A lower bacterial diversity than *S. avenae* was observed with an estimated mean Shannon diversity index of 0.07 (SD = 0.04) (Table 1). *B. aphidicola* was found in a percentage >98.5% in all DNA pools, however no additional bacteria were found in *R. padi* (Fig. 1). Finally, a low proportion of unassigned reads was detected among the six DNA pools (<0.01%), as well the proportion of unrepresented reads was found in an average of 0.24% of the total reads, being the highest proportion of unrepresented reads detected in the DNA pool RP-1 (1.3% of the total of reads) (Fig. 1). Sequencing data generated on Illumina were submitted to GenBank (accession numbers: MG958610, MG958611, MG958612 MG958613 and MG958614) (Data S2).

### 16S rRNA sequencing and phylogenetic analysis of *Pseudomonas* species

**Figure 2:**
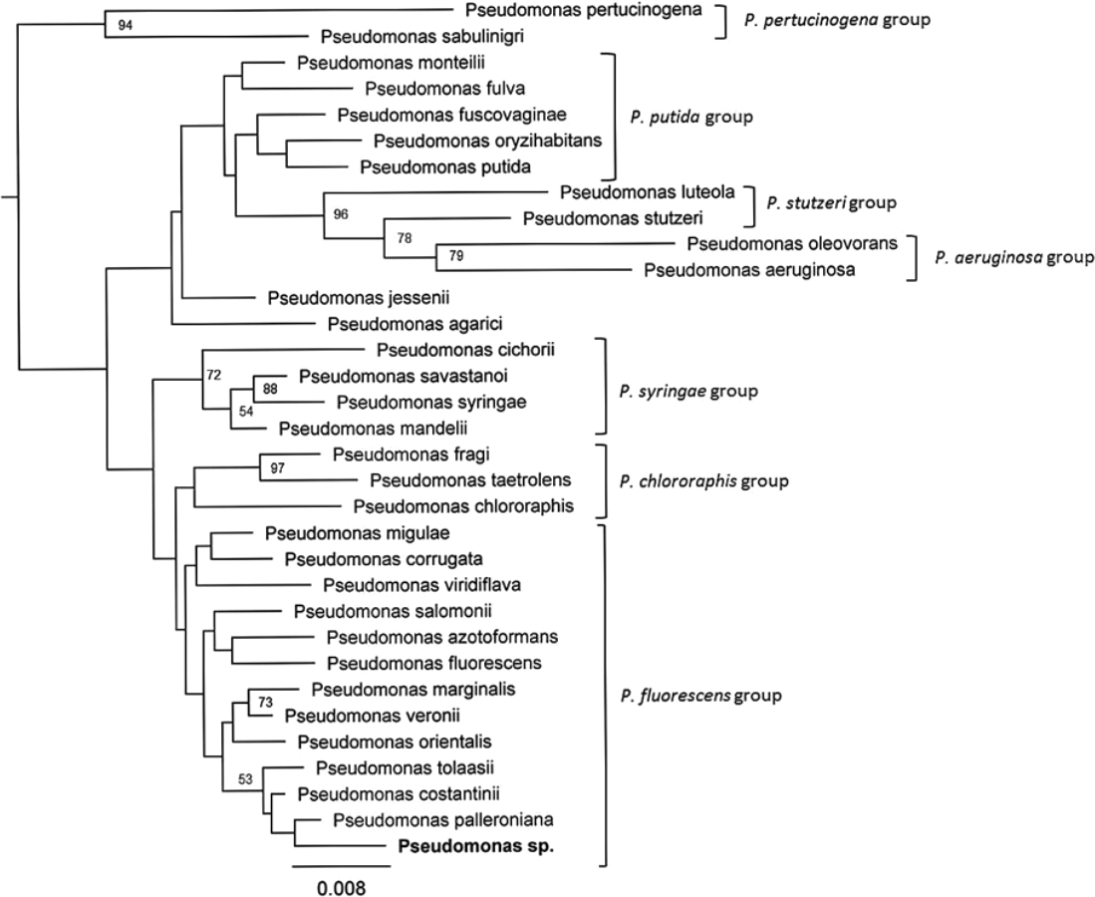
Neighbor-Joining tree based on 16S rRNA sequences of 32 known species of the genus *Pseudomonas*. *Pseudomonas* sp. corresponds to the sequence amplified from the aphid *S. avenae*.

Of the sequences generated for the 20 aphid samples of *S. avenae*, only one DNA sample corresponded to a *Pseudomonas* species (GenBank accession number MF536106). Sequences of the other DNA samples were observed as belonging to some of the other aphid secondary endosymbionts (*R. insecticola* and *H. defensa*), as it was identified by the 16S rRNA sequencing. The phylogenetic tree constructed show the seven clusters previously described for the genus *Pseudomonas* (Fig. 2). The *Pseudomonas* sequence generated from aphid DNA was located into the “*P. fluorescens* group”, being closely related to *Pseudomonas palleroniana* with an identity percentage >95% (Fig. 2).

## Discussion

### Secondary endosymbionts in the introduced aphid populations

A low bacterial diversity in the introduced populations of the cereal aphids *S. avenae* and *R. padi* was revealed by 16S rRNA amplicon sequencing in Chile. Gammaproteobacteria was the most common class identified and as expected the aphid primary endosymbiont, *B. aphidicola*, was the most common bacterial species detected in *S. avenae* and *R. padi*. In all DNA pools of both aphid species, *Buchnera* made up a large percentage of all of the reads (ranged between 84.4% and 99% respectively). In contrast to our systems, a greater diversity of secondary endosymbionts can be found in other aphid species (Zytynska & Weisser, 2016). For instance, the well-studied pea aphid, *A. pisum*, hosts at least eight secondary endosymbionts (*Serratia symbiotica*, *R. insecticola*, *H. defensa*, *Rickettsiella*, PAXS, *Spiroplasma, Rickettsia* and *Wolbachia*) that are highly abundant according to two 16 rRNA amplicon sequencing studies (Russell et al., 2013; Gauthier et al., 2015). A similar study in several aphid species of the genus *Cinara* has detected the presence of four dominant secondary endosymbionts (*S. symbiotica*, *H. defensa*, *R. insecticola* and *Wolbachia*) (Jousselin et al., 2016). Our results show an absence of secondary endosymbionts in the introduced populations of the aphid *R. padi*. Whilst the diversity of secondary endosymbionts observed in the aphid *S. avenae* was low; only two well-known aphid endosymbionts were encountered, *R. insecticola* and *H. defensa.* A similar bacterial diversity has been found in other geographic populations of these aphid species. For instance, aphid samples screened for a set of endosymbionts (*H. defensa*, *R. insecticola*, *S. symbiotica*, *Rickettsia*, *Rickettsiella* and *Spiroplasma*) have found only three secondary endosymbionts (*H. defensa*, *R. insecticola* and *S. symbiotica*) in the native range of *S. avenae* (U.K. and Germany) (Łukasik et al., 2013; Henry et al., 2015; Alkhedir et al., 2015). In particular, a positive association between *H. defensa* and *S. avenae* was found, being this the most common endosymbiont followed by *R. insecticola*, whilst *S. symbiotica* was reported in a lower frequency (≤6%) in the aphid populations (Łukasik et al., 2013; Henry et al., 2015). Differently, higher infection rates of *R. insecticola* and *S. symbiotica* were found in Chinese populations of *S. avenae* (Luo et al., 2016), as well a high prevalence of *R. insecticola* (75% of infected aphids) was found in introduced populations of *S. avenae* in Morocco (Fakhour et al., 2018). In this study, we found that *R. insecticola* was the dominant secondary endosymbiont in *S. avenae*, while *H. defensa* was observed at lower prevalence among DNA samples studied. However, the read abundance should be interpreted carefully when it is used as an estimate of the infection frequency of endosymbionts, because PCR amplification bias can be introduced by primer specificity (Klindworth et al., 2012). Despite this, our results from the deep sequencing of 16S rRNA gene are consistent with previous PCR-based studies on Chilean populations of *S. avenae*, in which ∼50% of the aphids harbored *R. insecticola* and a lower proportion of aphids harbored *H. defensa* (between 4% and 15%) (Sepúlveda et al., 2016; Zepeda-Paulo, Villegas & Lavandero, 2017), suggesting that the aphid-endosymbiont associations can vary across geographic range of aphid species.

Secondary endosymbionts make up an important component of the bacterial community of aphids and several studies have indicated that they have important effects on the host phenotype. Specifically, aphid secondary endosymbionts can protect the host from natural enemies, can provide tolerance to heat shock and can facilitate the colonization of new host plants (Oliver et al., 2010). Although recent studies have not found evidence that the endosymbionts *R. insecticola* nor *H. defensa* can confer defense against parasitoid wasps in *S. avenae* (Łukasik et al., 2013; Zepeda-Paulo, Villegas & Lavandero, 2017), at least one strain of *R. insecticola* has shown to provide protection to *S. avenae* against the pathogenic fungus *Pandora neoaphidis* (Łukasik et al., 2015). This symbiont-mediated advantage could explain the higher prevalence of *R. insecticola* in the populations of *S. avenae* here studied; however, this is not consistent with the lower prevalence of this endosymbiont reported in native regions of *S. avenae.* An explanation for this observation could be the founder effect and drift experienced by aphid populations introduced in a new region (Desneux et al., 2018). During the invasive process only a subset of symbiont-harboring aphid clones may have been introduced from the native regions, resulting in particular aphid—endosymbiont associations in the novel established populations. Indeed, variation in the associations between aphid clones and endosymbionts can be found in field populations, suggesting that they could be relevant for understanding of aphid—symbiont populations dynamics (Zepeda-Paulo, Villegas & Lavandero, 2017). In addition, we cannot rule out the effect of sampling method (e.g., number and distribution of sampling in a season) on the infection rates observed in aphid populations, since the frequency of endosymbionts can increase and/or fluctuate during the course of a season (Henry et al., 2015). In this regard, our aphid sampling would be considered representative of the endosymbiont diversity, as it was performed during the period of highest abundance of aphids (Raymond, Ortiz-Martínez & Lavandero, 2015; Ortiz-Martínez & Lavandero, 2018) and endosymbionts of the populations in the field (F Zepeda-Paulo & B Lavandero, 2018, unpublished data).

Unlike *S. avenae*, there is little knowledge on the diversity of bacterial endosymbionts in the aphid *R. padi*. Despite this, the existing data are consistent with our results in show an absence of secondary endosymbionts in aphid samples from the native range of *R. padi* (Europe) analyzed using species-specific primers developed for three aphid endosymbionts (*H. defensa*, *R. insecticola* and *S. symbiotica*) (Henry et al., 2015; Desneux et al., 2018) nor their introduced range (Morocco) using 16S rRNA gene sequencing (Fakhour et al., 2018). The bacterial diversity could be non-randomly distributed throughout host species. In this sense, it has been raised that the prevalence of secondary endosymbionts in a particular insect host may depend on the balance between the costs and benefits of harboring symbionts (Oliver, Smith & Russell, 2014). Indeed, the lack of an important protective phenotype providing direct benefits, fitness costs on symbiont-harboring host and the transmission rates of endosymbionts are some of the factors that could explain the low occurrence of endosymbionts in a particular host species (Oliver, Smith & Russell, 2014; Dykstra et al., 2014). Another factor that may influence bacterial diversity of aphids are the symbiont-symbiont interactions, such as competition between primary and secondary endosymbionts. Regarding this, several studies have shown that the density of the aphid primary endosymbiont, *B. aphidicola,* could be affected by the coexistence with secondary endosymbionts in the same host (Koga, Tsuchida & Fukatsu, 2003; Sakurai et al., 2005; Leclair et al., 2017). A negative effect on *Buchnera* abundance may be detrimental to the fitness of aphids and could significantly affect some aphid species. Aphids species can vary in their ability to increase the amino acid concentration in the phloem, in response to chlorotic damage induced by them (Sandström, Telang & Moran, 2000). This increase may reduce the nutritional dependence of aphids on *Buchnera* for the synthesis of essential amino acids, which could affect the aphid-symbiont associations. For instance, *R. padi* could show a high dependence on *Buchnera* for the synthesis of essential amino acids, since this does not affect the phloem composition of the host plant, compared to a higher amino acid concentration induced by other aphid species (Sandström & Moran, 1999; Sandström, Telang & Moran, 2000). A greater dependence in *Buchnera* could limit the infection of secondary endosymbionts, if they affect the abundance of the primary endosymbiont of hosts and thus explain the absence of secondary endosymbionts in some aphid species. However, the association between *Buchnera*-dependent aphids and the prevalence of secondary endosymbionts still have to be studied for a better understanding of the role of symbiont–symbiont interactions on the bacterial diversity of aphid species.

### Presence of *Pseudomonas* sp. in cereal aphids

In addition to the most common aphid endosymbionts, the results from 16S rRNA sequencing showed the occurrence of *Pseudomonas* sp. in two DNA pools analyzed of the aphid *S. avenae*. However, of the sequences generated for 20 aphid samples of *S. avenae*, only one DNA sample corresponded to a *Pseudomonas* species. The phylogenetic analysis incorporating known *Pseudomonas* sequences showed clustering with the “*P. fluorescens* group”; the *Pseudomonas* sp. sequence generated here was closely related to the bacteria *P. palleroniana* and *P. tolassi*. These bacterial species are known phytopathogenic Pseudomonads, which have been found in rice (*Oryza sativa*) and garlic (*Allium sativum*), respectively (Gardan et al., 2002; Höfte & De Vos, 2007). Others studies based on 16 rRNA amplicon sequencing have identified phytopathogenic *Pseudomonas* sp. in the pea aphid (*Pseudomonas syringae*) and *R. padi* (*P. viridiflava* and *P. veronii*) (Gauthier et al., 2015). Moreover, the pea aphid has previously proven capable of acting as both a vector and a non-plant host for *P. syringae* (Stavrinides, McCloskey & Ochman, 2009). Some strains of *P. syringae* could be pathogenic to aphids causing death by bacterial sepsis (Stavrinides, McCloskey & Ochman, 2009; Hendry, Clark & Baltrus, 2016). The finding of *Pseudomonas* sp. in different aphid species suggests that these types of phytopathogen-vector associations may be more common than previously thought among aphid species. Secondary endosymbionts can also influence the interactions between phytopathogens and insects. Hendry, Hunter & Baltrus (2014) reported that secondary endosymbionts can influence interactions between whiteflies and the phytopathogen (*P. syringae*); whiteflies harboring *Rickettsia* have decreased their mortality from *P. syringae* (Hendry, Hunter & Baltrus, 2014). This latter finding might suggest that similar interactions among endosymbiotic and phytopathogenic bacteria may also occur in other host insects (Gonzalez et al., 2016). However, there are currently no studies on the extent of phytopathogen-vector/host associations or the effect of secondary endosymbionts on the interactions between aphids and phytopathogenic bacteria.

## Conclusions

The study presented employing 16S rRNA gene sequencing indicates that the bacterial diversity of the introduced populations of the aphid pests, *S. avenae* and *R. padi*, is low. A similar endosymbiont diversity has been reported for both aphid species in their native range. However, variation in the secondary endosymbiont infection could be observed among the introduced and native populations of the aphid *S. avenae,* indicating that aphid-endosymbiont associations can vary across the geographic range of an aphid species. Our results showed that *R. insecticola* was the dominant secondary endosymbiont of the introduced populations*,* while this endosymbiont could be less important in the native range of *S. avenae*; where *H. defensa* is the most common endosymbiont reported*.* Interestingly, the presence of a *Pseudomonas* sp. closely related to phytopathogenic Pseudomonad species was detected in the aphid samples. As has been observed for other aphids, the detection of *Pseudomonas* sp. could suggest that aphids can act as a potential vector of phytopathogenic bacteria. However, further studies are necessary to determine the role of aphid species as vectors and/or alternative hosts of important phytopathogenic bacteria.

##  Supplemental Information

10.7717/peerj.4725/supp-1 Data S1 Accession numbers and references for the *Pseudomonas* 16S rRNA sequences used to construct the phylogenetic tree

10.7717/peerj.4725/supp-2 Data S2 Summary of total number of reads per aphid samples of *S. avenae* and *R. padi* for representative OTUs and GenBank accession number of representative sequences

## Additional Information and Declarations

### Field Study Permissions

The following information was supplied relating to field study approvals (i.e., approving body and any reference numbers): Field experiments were approved by the Ethics committee of the Universidad de Talca (project number: 3140299).

### Data Availability

The following information was supplied regarding data availability: GenBank: MF536106 and MG958610–MG958614.

## References

[ref-1] Alkhedir H, Karlovsky P, Mashaly AMA, Vidal S (2015). Phylogenetic relationships of the symbiotic bacteria in the aphid *Sitobion avenae* (Hemiptera: Aphididae). Environmental Entomology.

[ref-2] Anzai Y, Kim H, Park J-Y, Wakabayashi H, Oyaizu H (2000). Phylogenetic affiliation of the pseudomonads based on 16S rRNA sequence. International Journal of Systematic and Evolutionary Microbiology.

[ref-3] Baumann P (2005). Biology of bacteriocyte-associated endosymbionts of plant sap-sucking insects. Annual Review of Microbiology.

[ref-4] Bolger AM, Lohse M, Usadel B (2014). Trimmomatic: a flexible trimmer for Illumina sequence data. Bioinformatics.

[ref-5] Caporaso JG, Kuczynski J, Stombaugh J, Bittinger K, Bushman FD, Costello EK, Fierer N, Pena AG, Goodrich JK, Gordon JI, Huttley GA (2010). QIIME allows analysis of high-throughput community sequencing data. Nature Methods.

[ref-6] Dedryver C-A, Le Ralec A, Fabre F (2010). The conflicting relationships between aphids and men: a review of aphid damage and control strategies. Comptes Rendus Biologies.

[ref-7] Desneux N, Asplen MK, Brady CM, Heimpel GE, Hopper KR, Luo C, Monticelli L, Oliver KM, White JA (2018). Intraspecific variation in facultative symbiont infection among native and exotic pest populations: potential implications for biological control. Biological Control.

[ref-8] Douglas A (1998). Nutritional interactions in insect-microbial symbioses: aphids and their symbiotic bacteria *Buchnera*. Annual Review of Entomology.

[ref-9] Drummond A, Ashton B, Buxton S, Cheung M, Cooper A, Duran C, Field M, Heled J, Kearse M, Markowitz S, Moir R, Stones-Havas S, Sturrock S, Thierer T, Wilson A (2011).

[ref-10] Dykstra HR, Weldon SR, White JA, Hopper KR, Heimpel GE, Asplen MK, Oliver KM (2014). Factors limiting the spread of the protective symbiont *Hamiltonella defensa* in the aphid *Aphis craccivora*. Applied and Environmental Microbiology.

[ref-11] Fakhour S, Ambroise J, Renoz F, Foray V, Gala JL, Hance T (2018). A large-scale field study of bacterial communities in cereal aphid populations across Morocco. FEMS Microbiology Ecology.

[ref-12] Ferrari J, Scarborough CL, Godfray HCJ (2007). Genetic variation in the effect of a facultative symbiont on host-plant use by pea aphids. Oecologia.

[ref-13] Gardan L, Bella P, Meyer J-M, Christen R, Rott P, Achouak W, Samson R (2002). *Pseudomonas salomonii* sp. nov., pathogenic on garlic, and *Pseudomonas palleroniana* sp. nov., isolated from rice. International Journal of Systematic and Evolutionary Microbiology.

[ref-14] Gauthier J-P, Outreman Y, Mieuzet L, Simon J-C (2015). Bacterial communities associated with host-adapted populations of pea aphids revealed by deep sequencing of 16S ribosomal DNA. PLOS ONE.

[ref-15] Gibson CM, Hunter MS (2010). Extraordinarily widespread and fantastically complex: comparative biology of endosymbiotic bacterial and fungal mutualists of insects. Ecology Letters.

[ref-16] Gonzalez F, Tkaczuk C, Dinu MM, Fiedler Ż, Vidal S, Zchori-Fein E, Messelink GJ (2016). New opportunities for the integration of microorganisms into biological pest control systems in greenhouse crops. Journal of Pest Science.

[ref-17] Hendry TA, Clark KJ, Baltrus DA (2016). A highly infective plant-associated bacterium influences reproductive rates in pea aphids. Royal Society Open Science.

[ref-18] Hendry TA, Hunter MS, Baltrus DA (2014). The facultative symbiont Rickettsia protects an invasive whitefly against entomopathogenic *Pseudomonas syringae* strains. Applied and Environmental Microbiology.

[ref-19] Henry LM, Maiden MC, Ferrari J, Godfray HC (2015). Insect life history and the evolution of bacterial mutualism. Ecology Letters.

[ref-20] Höfte M, De Vos P, Gnanamanickam SS (2007). Plant pathogenic *Pseudomonas* species *Plant-associated bacteria*. Plant-associated bacteria.

[ref-21] Jaenike J, Unckless R, Cockburn SN, Boelio LM, Perlman SJ (2010). Adaptation via symbiosis: recent spread of a *Drosophila* defensive symbiont. Science.

[ref-22] Jiggins FM, Hurst GD (2011). Rapid insect evolution by symbiont transfer. Science.

[ref-23] Jousselin E, Clamens AL, Galan M, Bernard M, Maman S, Gschloessl B, Duport D, Meseguer AS, Calavro F, Coeur d’acier A (2016). Assessment of a 16S rRNA amplicon Illumina sequencing procedure for studying the microbiome of a symbiontrich aphid genus. Molecular Ecology Resources.

[ref-24] Klindworth A, Pruesse E, Schweer T, Peplies J, Quast C, Horn M, Glöckner FO (2012). Evaluation of general 16S ribosomal RNA gene PCR primers for classical and next-generation sequencing-based diversity studies. Nucleic Acids Research.

[ref-25] Koga R, Tsuchida T, Fukatsu T (2003). Changing partners in an obligate symbiosis: a facultative endosymbiont can compensate for loss of the essential endosymbiont *Buchnera* in an aphid. Proceedings of the Royal Society B: Biological Sciences.

[ref-26] Kunin V, Engelbrektson A, Ochman H, Hugenholtz P (2010). Wrinkles in the rare biosphere: pyrosequencing errors can lead to artificial inflation of diversity estimates. Environmental Microbiology.

[ref-27] Leclair M, Polin S, Jousseaume T, Simon JC, Sugio A, Morlière S, Fukatsu T, Tsuchida T, Outreman Y (2017). Consequences of coinfection with protective symbionts on the host phenotype and symbiont titres in the pea aphid system. Insect Science.

[ref-28] Łukasik P, Dawid MA, Ferrari J, Godfray HCJ (2013). The diversity and fitness effects of infection with facultative endosymbionts in the grain aphid, *Sitobion avenae*. Oecologia.

[ref-29] Łukasik P, Guo H, Asch M, Henry LM, Godfray HCJ, Ferrari J (2015). Horizontal transfer of facultative endosymbionts is limited by host relatedness. Evolution.

[ref-30] Luo C, Luo K, Hu ZQ, Tao YY, Zhao HY (2016). The infection frequencies and dynamics of three secondary endosymbionts in the laboratory environments on *Sitobion avenae* (Fabricius) as determined by long PCR. Journal of Asia-Pacific Entomology.

[ref-31] Masella AP, Bartram AK, Truszkowski JM, Brown DG, Neufeld JD (2012). PANDAseq: paired-end assembler for illumina sequences. BMC Bioinformatics.

[ref-32] Montllor CB, Maxmen A, Purcell AH (2002). Facultative bacterial endosymbionts benefit pea aphids *Acyrthosiphon pisum* under heat stress. Ecological Entomology.

[ref-33] Moran NA, McCutcheon JP, Nakabachi A (2008). Genomics and evolution of heritable bacterial symbionts. Annual Review of Genetics.

[ref-34] Nadarasah G, Stavrinides J (2011). Insects as alternative hosts for phytopathogenic bacteria. FEMS Microbiology Reviews.

[ref-35] Ng JC, Perry KL (2004). Transmission of plant viruses by aphid vectors. Molecular Plant Pathology.

[ref-36] Oliver KM, Degnan PH, Burke GR, Moran NA (2010). Facultative symbionts in aphids and the horizontal transfer of ecologically important traits. Annual Review of Entomology.

[ref-37] Oliver KM, Moran NA, Hunter MS (2005). Variation in resistance to parasitism in aphids is due to symbionts not host genotype. Proceedings of the National Academy of Sciences of the United States of America.

[ref-38] Oliver KM, Russell JA, Moran NA, Hunter MS (2003). Facultative bacterial symbionts in aphids confer resistance to parasitic wasps. Proceedings of the National Academy of Sciences of the United States of America.

[ref-39] Oliver KM, Smith AH, Russell JA (2014). Defensive symbiosis in the real world—advancing ecological studies of heritable, protective bacteria in aphids and beyond. Functional Ecology.

[ref-40] Ortiz-Martínez SA, Lavandero B (2018). The effect of landscape context on the biological control of *Sitobion avenae*: temporal partitioning response of natural enemy guilds. Journal of Pest Science.

[ref-41] Parker BJ, Spragg CJ, Altincicek B, Gerardo NM (2013). Symbiont-mediated protection against fungal pathogens in pea aphids: a role for pathogen specificity?. Applied and Environmental Microbiology.

[ref-42] Peccoud J, Bonhomme J, Mahéo F, Huerta M, Cosson O, Simon JC (2014). Inheritance patterns of secondary symbionts during sexual reproduction of pea aphid biotypes. Insect Science.

[ref-43] Raymond L, Ortiz-Martínez SA, Lavandero B (2015). Temporal variability of aphid biological control in contrasting landscape contexts. Biological Control.

[ref-44] Riesenfeld CS, Schloss PD, Handelsman J (2004). Metagenomics: genomic analysis of microbial communities. Annual Review of Genetics.

[ref-45] Russell JA, Moran NA (2005). Horizontal transfer of bacterial symbionts: heritability and fitness effects in a novel aphid host. Applied and Environmental Microbiology.

[ref-46] Russell JA, Weldon S, Smith AH, Kim KL, Hu Y, Łukasik P, Doll S, Anastopoulos I, Novin M, Oliver KM (2013). Uncovering symbiont-driven genetic diversity across North American pea aphids. Molecular Ecology.

[ref-47] Sakurai M, Koga R, Tsuchida T, Meng XY, Fukatsu T (2005). *Rickettsia* symbiont in the pea aphid *Acyrthosiphon pisum*: novel cellular tropism, effect on host fitness, and interaction with the essential symbiont Buchnera. Applied and Environmental Microbiology.

[ref-48] Salter SJ, Cox MJ, Turek EM, Calus ST, Cookson WO, Moffatt MF, Turner P, Parkhill J, Loman NJ, Walker AW (2014). Reagent and laboratory contamination can critically impact sequence-based microbiome analyses. BMC Biology.

[ref-49] Sandström J, Moran N (1999). How nutritionally imbalanced is phloem sap for aphids?.

[ref-50] Sandström JP, Russell JA, White JP, Moran NA (2001). Independent origins and horizontal transfer of bacterial symbionts of aphids. Molecular Ecology.

[ref-51] Sandström J, Telang A, Moran NA (2000). Nutritional enhancement of host plants by aphids—a comparison of three aphid species on grasses. Journal of Insect Physiology.

[ref-52] Scarborough CL, Ferrari J, Godfray H (2005). Aphid protected from pathogen by endosymbiont. Science.

[ref-53] Schmieder R, Edwards R (2011). Quality control and preprocessing of metagenomic datasets. Bioinformatics.

[ref-54] Sepúlveda DA, Zepeda-Paulo F, Ramírez CC, Lavandero B, Figueroa CC (2016). Diversity, frequency and geographic distribution of facultative bacterial endosymbionts in introduced aphid pests. Insect Science.

[ref-55] Simon JC, Rispe C, Sunnucks P (2002). Ecology and evolution of sex in aphids. Trends in Ecology & Evolution.

[ref-56] Simon J-C, Boutin S, Tsuchida T, Koga R, Le Gallic J-F, Frantz A, Outreman J, Fukatsu T (2011). Facultative symbiont infections affect aphid reproduction. PLOS ONE.

[ref-57] Stavrinides J,  McCloskey JK, Ochman H (2009). Pea aphid as both host and vector for the phytopathogenic bacterium *Pseudomonas syringae*. Applied and Environmental Microbiology.

[ref-58] Sunnucks P, Hales DF (1996). Numerous transposed sequences of mitochondrial cytochrome oxidase I-II in aphids of the genus *Sitobion* (Hemiptera: Aphididae). Molecular Biology and Evolution.

[ref-59] Toft C, Andersson SG (2010). Evolutionary microbial genomics: insights into bacterial host adaptation. Nature Reviews Genetics.

[ref-60] Tsuchida T, Koga R, Fukatsu T (2004). Host plant specialization governed by facultative symbiont. Science.

[ref-61] Tsuchida T, Koga R, Matsumoto S, Fukatsu T (2011). Interspecific symbiont transfection confers a novel ecological trait to the recipient insect. Biology Letters.

[ref-62] Van Emden HF, Harrington R (2017). Aphids as crop pests.

[ref-63] Vorburger C (2014). The evolutionary ecology of symbiont conferred resistance to parasitoids in aphids. Insect Science.

[ref-64] Vorburger C, Gehrer L, Rodriguez P (2009). A strain of the bacterial symbiont *Regiella insecticola* protects aphids against parasitoids. Biology Letters.

[ref-65] Werren JH, Baldo L, Clark ME (2008). *Wolbachia*: master manipulators of invertebrate biology. Nature Reviews Microbiology.

[ref-66] Zepeda-Paulo F, Villegas C, Lavandero B (2017). Host genotype-endosymbiont associations and their relationship with aphid parasitism at the field level. Ecological Entomology.

[ref-67] Zytynska SE, Weisser WW (2016). The natural occurrence of secondary bacterial symbionts in aphids. Ecological Entomology.

