# Supplementary material for: Low bacterial community diversity in two introduced aphid pests revealed with 16S rRNA amplicon sequencing
Source: PeerJ. 2018 May 7;6:e4725. doi: 10.7717/peerj.4725 (PMC5944429; doi:10.7717/peerj.4725)
Supplement: Data S1 [file peerj-06-4725-s001.docx]

Supplementary data 1. Accession numbers and references for the *Pseudomonas* 16S rRNA sequences used to construct the phylogenetic tree.

| ***Pseudomonas* species** | **Accession no.** | **Reference** |
| --- | --- | --- |
| *Pseudomonas pertucinogena* | AB021380 | Anzai et al. (2000) |
| *Pseudomonas sabulinigri* | EU14352 | Kim et al. (2009) |
| *Pseudomonas anguilliseptica* | AB021376 | Kersters et al. (1996) |
| *Pseudomonas oleovorans* | D84018 | Kersters et al. (1996) |
| *Pseudomonas aeruginosa* | Z76651 | Kersters et al. (1996) |
| *Pseudomonas balearica* | U26418 | Kersters et al. (1996) |
| *Pseudomonas luteola* | D84002 | Anzai et al. (2000) |
| *Pseudomonas stutzeri* | U26262 | Kersters et al. (1996) |
| *Pseudomonas putida* | D84020 | Kersters et al. (1996) |
| *Pseudomonas fulva* | D84015 | Anzai et al. (1997) |
| *Pseudomonas monteilii* | AB021409‹ | Anzai et al. (2000) |
| *Pseudomonas oryzihabitans* | D84004 | Anzai et al. (1997) |
| *Pseudomonas viridiflava* | Z76671 | Kersters et al. (1996) |
| *Pseudomonas syringae* | D84026 | Kersters et al. (1996) |
| *Pseudomonas savastanoi* | AB021402‹ | Kersters et al. (1996) |
| *Pseudomonas cichorii* | AB021398 | Kersters et al. (1996) |
| *Pseudomonas chlororaphis* | D84011 | Kersters et al. (1996) |
| *Pseudomonas fragi* | AB021413Œ | Kersters et al. (1996) |
| *Pseudomonas taetrolens* | D84027 | Kersters et al. (1996) |
| *Pseudomonas fuscovaginae* | AB021381‹ | Kersters et al. (1996) |
| *Pseudomonas jessenii'* | AF068259 | Anzai et al (2000) |
| *Pseudomonas agarici* | D84005 | Kersters et al. (1996) |
| *Pseudomonas fluorescens* | D84013 | Kersters et al. (1996) |
| *Pseudomonas corrugata* | D84012 | Kersters et al. (1996) |
| *Pseudomonas migulae* | AF074383 | Anzai et al. (2000) |
| *Pseudomonas mandelii* | AF058286 | Anzai et al. (2000) |
| *Pseudomonas marginalis* | AB021401‹ | Kersters et al. (1996) |
| *Pseudomonas orientalis* | AF064457 | Anzai et al. (2000) |
| *Pseudomonas tolaasii* | D84028 | Kersters et al. (1996) |
| *Pseudomonas azotoformans* | D84009 | Anzai et al. (1997) |
| *Pseudomonas veronii* | AB021411‹ | Anzai et al. (2000) |
| *Pseudomonas constantinii* | AF374472 | Munsch et al. (2002) |
| *Pseudomonas pelleroriana* | AY091527 | Gardan et al. (2002) |
| *Pseudomonas salomonii* | NR_029051 | Gardan et al. (2002) |
