# Supplementary material for: Low bacterial community diversity in two introduced aphid pests revealed with 16S rRNA amplicon sequencing
Source: PeerJ. 2018 May 7;6:e4725. doi: 10.7717/peerj.4725 (PMC5944429; doi:10.7717/peerj.4725)
Supplement: Data S2 [file peerj-06-4725-s002.docx]

| OTU ID | *S. avenae* samples | | | | Accession number | *R. padi* samples | | | | | | Accession number |
| --- | --- | --- | --- | --- | --- | --- | --- | --- | --- | --- | --- | --- |
|  | SA-1 | SA-2 | SA-3 | SA-4 |  | RP-1 | RP-2 | RP-3 | RP-4 | RP-5 | RP-6 |  |
| Buchnera aphidicola | 146434 | 382329 | 522510 | 378472 | MG958611 | 207358 | 371927,00 | 402438 | 351251 | 556187 | 202961 | MG958610 |
| Regiella insecticola | 11569 | 44680 | 75296 | 26328 | MG958612 | 0 | 0 | 0 | 0 | 0 | 0 |  |
| Hamiltonella defensa | 7749 | 124 | 7860 | 57 | MG958613 | 0 | 0 | 0 | 0 | 0 | 0 |  |
| Pseudomonas sp. | 48877 | 5 | 9 | 7233 | MG958614 | 0 | 0 | 0 | 0 | 0 | 0 |  |
| Unassigned | 1509 | 2270 | 3355 | 1977 |  | 2 | 21 | 16 | 6 | 27 | 3 |  |
| Unrepresented | 16596 | 928 | 3335 | 3709 |  | 2759 | 65 | 61 | 79 | 374 | 69 |  |
| Total | 232734 | 430336 | 612365 | 417776 |  | 210119 | 372013 | 402515 | 351336 | 556588 | 203033 |  |

Tabla S2 Summary of total number of reads per aphid samples of *S. avenae* and *R. padi* for representative OTUs and GenBank accession number of representative sequences.
